# Genome Sequences of 17 Strains from Eight Races of Xanthomonas campestris pv. campestris

**DOI:** 10.1128/mra.00279-22

**Published:** 2022-06-13

**Authors:** Caroline Bellenot, Sébastien Carrère, Carine Gris, Laurent D. Noël, Matthieu Arlat

**Affiliations:** a INRAE, UMR 441, Laboratoire des Interactions Plantes-Microbes-Environnement, Castanet-Tolosan, France; b CNRS, UMR 2594, Laboratoire des Interactions Plantes-Microbes-Environnement, Castanet-Tolosan, France; c Université de Toulouse, Université Paul Sabatier, Toulouse, France; University of Arizona

## Abstract

Xanthomonas campestris pv. campestris is a group of phytopathogenic bacteria causing black rot disease on Brassicaceae crops. Here, we report on draft genome sequences of 17 strains representing eight of nine known races of this pathogen, including the pathotype strain CFBP 6865.

## ANNOUNCEMENT

Genomic analysis is a powerful approach to study the diversity and evolution of virulence determinants of pathogenic bacteria (1, 2). Xanthomonas campestris pv. campestris is the causal agent of black rot disease, affecting many crop plants from the Brassicaceae family (3). X. campestris pv. campestris strains have been organized into nine races based on symptoms caused on a set of host and nonhost plants (3). Amplified fragment length polymorphism (AFLP) allowed the classification of X. campestris pv. campestris strains into seven genomic clades (clades A to G) (4), which is not congruent with the race classification. Here, we present the genome sequences of 17 X. campestris pv. campestris strains (Table 1), including the pathotype strain CFBP 6865. These strains were isolated from different crop varieties or subspecies of Brassica oleracea and Brassica rapa between 1958 and 2002, in Australia, Belgium, China, France, or Germany. They belong to four of the seven genomic clades and are representative of eight of the nine known races. All of these strains were obtained from the International Centre for Microbial Resources-French Collection for Plant-Associated Bacteria (CIRM-CFBP), National Research Institute for Agriculture, Food, and the Environment (INRAE).

**TABLE 1:** Description of the Xanthomonas campestris pv. campestris strains reported and statistics for the sequencing and assembly of their genomes

Strain	CFBP designation^*a*^	Clade^*b*^	Ra ce^*c*^	Date of isolat ion	Host	Country of isolation	No. of rea ds	Genome size (bp)	Genome coverage (×)	No. of contigs	*N*_50_ (bp)	GC content (%)	BUSCO completeness (%)	No. of coding sequences	GenBank accession no.	SRA accession no.	DOI no.
147	CFBP 7156		4		*Brassica oleracea*		10,035,656	5,060,825	320	141	80,681	65.0	99.3	4,305	AVEU00000000.1/	SRP322641	10.25794/reference/um8maue7
CFBP 1119	CFBP 1119	A	1	1967	*Brassica oleracea* var. botrytis	France	35,046,928	5,008,837	1,063	74	183,459	64.6	99.9	4,220	CBVE000000000.1/	SRP322638	10.25794/reference/njvvm7l0
CFBP 1124	CFBP 1124	C	8	1967	*Brassica oleracea* var. botrytis	France	7,211,603	4,997,962	240	103	149,591	65.6	99.7	4,436	AVEV00000000.1/	SRP322639	10.25794/reference/yih1qnob
CFBP 1712	CFBP 1712	E	5	1975	*Brassica oleracea* cv. Capitata	France	17,215,274	5,015,961	553	129	79,498	64.9	98.6	4,461	AVDC00000000.1/	SRP322649	10.25794/reference/dnvsfr18
CFBP 1713	CFBP 1713	E	5	1975	*Brassica oleracea* var. *botrytis*	France	18,408,662	5,041,610	739	89	151,632	64.4	99.4	4,491	AVDM00000000.1/	SRP322642	10.25794/reference/rmbgyely
CFBP 4953	CFBP 4953		7	1999	*Brassica oleracea* var. botrytis cv. Cortes	Belgium	32,918,799	5,052,992	991	83	163,533	64.7	99.9	4,562	CBVF000000000.1/	SRP322648	10.25794/reference/nnnw_mqq
CFBP 4954	CFBP 4954		6	1999	*Brassica oleracea* var. botrytis cv. Aviso	Belgium	6,433,757	5,073,734	204	149	66,639	65.1	99.4	4,496	AVDE00000000.1/	SRP322643	10.25794/reference/fkxlurkw
CFBP 4955	CFBP 4955	E	9	1999	*Brassica oleracea* var. botrytis cv. Aviso	Belgium	4,239,839	5,136,167	138	142	84,609	65.1	99.7	4,600	AVDF00000000.1/	SRP322645	10.25794/reference/c02vneut
CFBP 4956	CFBP 4956		4	1999	*Brassica oleracea* var. botrytis cv. Spacestar	Belgium	37,480,089	4,965,880	1,148	125	109,963	64.5	99.8	4,396	CBVG000000000.1/	SRP322646	10.25794/reference/rx9gmcmt
CFBP 5130	CFBP 5130	A	7		*Brassica* sp.		9,232,935	5,056,538	295	129	93,783	64.9	99.7	4,561	AVDG00000000.1/	SRP322647	10.25794/reference/_016t91q
CFBP 5683	CFBP 5683	E	3	1979	*Brassica oleracea*	France	2,579,478	5,030,566	82	184	51,279	65.4	98.8	4,492	AVDH00000000.1/	SRP322644	10.25794/reference/0sr1ne3k
CFBP 6863	CFBP 6863		9	1958	*Brassica oleracea* var. botrytis	Germany	32,059,044	5,001,025	975	94	173,153	64.5	99.7	4,458	CBVH000000000.1/	SRP322661	10.25794/reference/shkh_1dj
CFBP 6865R^*d*^	CFBP 6865		5	1975	*Brassica oleracea* var. capitata	Australia	16,397,066	5,166,940	916	24	415,646	63.7	99.6	4,657	AVDT00000000.1/	SRX11066177	10.25794/reference/uce57zl_
CN01	CFBP 8237	A	1	2002	*Brassica rapa* subsp. *pekinensis*	China	20,801,984	5,030,897	836	75	179,901	64.3	99.9	4,543	AVDN00000000.1/	SRP322694	10.25794/reference/zzeitq6q
CN10	CFBP 8244	B	7	2002	*Brassica rapa* subsp. *pekinensis*	China	19,211,549	4,958,955	784	101	124,175	64.5	99.5	4,411	AVDR00000000.1/	SRP322708	10.25794/reference/h5nghmsn
CN19		B	4			China	32,316,246	4,958,083	992	113	115,047	64.4	99.5	4,422	CBVC000000000.1/	SRP322706	10.25794/reference/ez20luoj
CN20	CFBP 8251		1	2002	*Brassica oleracea* var. alboglabra	China	31,554,774	5,084,276	944	115	116,198	64.5	99.6	4,551	CBVD000000000.1/	SRP322707	10.25794/reference/e3ex38nh

a Strains are available in the CIRM-CFBP (www.cirm-cfbp.fr/page/Home).

b Clades based on AFLP of X. campestris pv. campestris strains reported by Guy et al. (4).

c Races as described previously by Vicente and Holub (3).

d Pathotype strain.

The X. campestris pv. campestris strains were grown overnight in MOKA-rich medium (4 g/L yeast extract, 8 g/L Casamino Acids, 2 g/L K_2_HPO_4_, and 0.3 g/L MgSO_4_·7H_2_O) at 28°C. From these cultures, genomic DNA was extracted with a Wizard genomic DNA purification kit (Promega). Genomic DNA libraries were prepared using a NEXTflex PCR-free DNA-sequencing kit (PerkinElmer) for most strains and a NEBNext kit (New England BioLabs) for strains CFBP 119, CFBP 4953, CFBP 4956, CFBP 6863, CN19, and CN20. All libraries were constructed following the manufacturers’ instructions. X. campestris pv. campestris total genomic DNA (including chromosome and plasmids) was sequenced on an Illumina HiSeq 2000 platform (2 × 101-bp paired-end reads) or a Genome Analyzer_IIx_ system (2 × 76-bp paired-end reads) (for strains CFBP 119, CFBP 4953, CFBP 4956, CFBP 6863, CN19, and CN20). Several assemblies were produced using the SOAPdenovo assembler (5) with incremental *k*-mer sizes (starting with a *k*-mer length corresponding to one-third of the read length, to a maximum of 99 nucleotides [nt], with a step of 4 nt). The resulting contigs were scaffolded using the Velvet assembler (6) (parameters: –cov_cutoff 5 –min_contig_length 100 –max_divergence 0.05 –exportFiltered yes –exp_cov auto) using the same *k*-mer range. A final round with SOAPGapCloser (parameter: –p 31) was performed to fill in the gaps, and the remaining redundancy (misassembled contigs) was removed using MegaBLAST-based identification. Genome sequences were annotated using the default parameters of the EuGene-P annotation pipeline to identify RNAs and protein-coding genes (7). The completeness of the genomes was assessed with BUSCO v5.1.2 (default parameters), using the xanthomonadales_odb10 data set (8, 9) (Table 1).

### Data availability.

The genome sequences were deposited in GenBank, and the raw reads were deposited in the Sequence Read Archive (SRA); their accession numbers are listed in Table 1. The genome annotations are available under the DOI numbers provided in Table 1.
